# The CONSENSUS study: protocol for a mixed methods study to establish which outcomes should be included in a core outcome set for oropharyngeal cancer

**DOI:** 10.1186/1745-6215-15-168

**Published:** 2014-05-13

**Authors:** Aoife MI Waters, Catrin Tudur Smith, Bridget Young, Terry M Jones

**Affiliations:** 1 Department of Biostatistics, University of Liverpool, 1st Floor Duncan Building, Daulby Street, Liverpool L69 3GA, UK; 2 Department of Molecular and Clinical Cancer Medicine, Liverpool Cancer Research UK Centre, University of Liverpool, 200 London Road, Liverpool L3 9TA, UK; 3 Department of Otolaryngology - Head and Neck Surgery, Aintree University Hospitals NHS Foundation Trust, Liverpool, UK; 4 Department of Psychological Sciences, University of Liverpool, Whelan Building, Brownlow Hill, Liverpool L69 3GB, UK

**Keywords:** Core outcome set, Consensus, Delphi, Oropharyngeal cancer, Head and neck cancer

## Abstract

**Background:**

The incidence of oropharyngeal cancer is increasing in the developed world. This has led to a large rise in research activity and clinical trials in this area, yet there is no consensus on which outcomes should be measured. As a result, the outcomes measured often differ between trials of comparable interventions, making the combination or comparison of results between trials impossible. Outcomes may also be ‘cherry-picked’, such that favourable results are reported, and less favourable results withheld. The development of a minimum outcome reporting standard, known as a core outcome set, goes some way to addressing these problems. Core outcome sets are ideally developed using a patient-centred approach so that the outcomes measured are relevant to patients and clinical practice. Core outcome sets drive up the quality and relevance of research by ensuring that the right outcomes are consistently measured and reported in trials in specific areas of health or healthcare.

**Methods/Design:**

This is a mixed methods study involving three phases to develop a core outcome set for oropharyngeal cancer clinical trials. Firstly, a systematic review will establish which outcomes are measured in published oropharyngeal cancer randomised controlled trials (RCTs). Secondly, qualitative interviews with patients and carers in the UK and the USA will aim to establish which outcomes are important to these stakeholders. Data from these first two stages will be used to develop a comprehensive list of outcomes to be considered for inclusion in the core outcome set. In the third stage, patients and clinicians will participate in an iterative consensus exercise known as a Delphi study to refine the contents of the core outcome set. This protocol lays out the methodology to be implemented in the CONSENSUS study.

**Discussion:**

A core outcome set defines a minimum outcome reporting standard for clinical trials in a particular area of health or healthcare. Its consistent implementation in oropharyngeal cancer clinical trials will improve the quality and relevance of research.

**Trials and registration:**

This study is registered at the National Institute for Health Research (NIHR) Clinical Research Network (CRN) portfolio, ID 13823 (17 January 2013).

## Background

Around 1,500 cases of oropharyngeal squamous cell carcinoma (OPSCC) are diagnosed in the UK every year [1]. Treatment relies on radiotherapy (RT), surgery with or without post-operative RT or cisplatin-based chemoradiotherapy (CRT), CRT alone or following induction chemotherapy. Increasingly, small molecule adjuvants are also being used. Multi-modality therapy is associated, to varying degrees, with long-term deficits in speech and swallowing function, cosmesis and health-related quality of life.

While the incidence of squamous cancers at most sub-sites of the head and neck is decreasing, the incidence of OPSCC has doubled in the last decade; and this trend is seen throughout the developed world [2,3]. Oncogenic human papillomavirus type 16 (HPV-16) has been established as the causative agent and has given rise to a clinicopathologically distinct form of OPSCC that occurs in a younger patient population and is associated with improved survival outcomes, compared with HPV-negative disease [4,5].

Contemporary treatments for HPV-positive and HPV-negative OPSCC are, however, still the same and treatment survivors suffer from long-term sequelae of multi-modality therapy, to varying degrees. Clinical trials in HPV-positive disease are largely focused on deintensifying treatment to improve functional outcomes whilst maintaining the advantageous survival outcomes; however, current research in HPV-negative disease remains focused on improving survival outcomes, with less apparent focus on reducing toxicity and improving functional outcomes. This is largely due to the fact that overall survival rates for HPV-negative disease have remained stubbornly resistant to improvement for many decades.

Despite the fact that available treatments have a significant impact on functioning and quality of life, such outcomes are inconsistently measured and reported between trials. Additionally, there is no standardisation of outcome selection and reporting, even among trials of comparable interventions. This reduces the amount of data contributable for meta-analyses, leading to difficulties in interpreting treatment effect and in making evidence-based healthcare decisions. The development of a minimum outcome reporting standard for OPSCC clinical trials, known as a core outcome set, is one method proposed to address these problems.

A core outcome set defines the outcomes that should be consistently measured and reported in clinical trials in a specific area of health or healthcare. They are developed using consensus methods involving major stakeholders, such as patients and healthcare professionals, to ensure that the outcomes included are clinically relevant and therefore ‘core’.

The existence of a core outcome set does not mean that only these outcomes should be measured; however, if a minimum outcome reporting standard is adhered to, then there will be greater consistency of reporting in clinical trials and a greater body of evidence to contribute to meta-analyses to inform healthcare decisions. Additionally, the risk of outcome reporting bias is lessened by ensuring that outcomes are consistently measured and reported.

The earliest efforts to improve outcome measurement in clinical trials were made by the Outcome Measures in Rheumatology (OMERACT) collaboration in the early 1990s [6]. This international network developed core sets of measures for most of the major rheumatological conditions, and an observational review by Kirkham *et al*. demonstrated an increase in the consistency of outcome reporting across clinical trials in rheumatoid arthritis (RA) in the years following publication of the RA core outcome set [7].

The OMERACT collaboration has actively involved patients in discussions about which outcomes to measure in trials since 2002 [8]. Patient involvement was first proposed at the OMERACT meeting in 2000 when participants considered what might be a ‘clinically important change’ in response to treatment. It was realised that the perspectives of patients are important in developing core outcome sets, and they have been actively involved in, and significantly contributed to, all subsequent OMERACT meetings. Patients have enriched the OMERACT research agenda, provided insights into patient participation in research and stimulated patient involvement in health outcomes research more broadly [9].

Other bodies are now recognising the importance of involving patients in trial research. INVOLVE, the national advisory group for the promotion and advancement of public involvement in NHS, public health and social care research, promotes the involvement of patients and the public in discussions about clinical trials because ‘they are the participants in trials and ultimately the people for whom research is aimed to benefit’ [10]. The Core Outcome Measures in Effectiveness Trials (COMET) Initiative advocates the involvement of patients and the public in decisions about which outcomes should be included in core outcome sets in specific areas of health or healthcare.

The measurement of patient-reported outcomes (PROs) in clinical trials has increased substantially in the last 20 years [11]. These subjective measures help evaluate the burden of disease and treatment from the patient’s perspective. The CONSORT group have recently published a PRO extension to their guidance that aims to improve the reporting of PROs in trials to facilitate the use of results in informing clinical practice and health policy [12].

Core outcome sets are now in development in a number of clinical areas and their use is advocated, in the UK, by the National Institute for Health Research (NIHR) Health Technology Assessment (HTA) and the Cochrane Collaboration [13,14]. Core outcome sets will only influence the evidence base if they are actually implemented, and as core outcome set developers we must therefore actively engage with trialists, Cochrane Review Groups, clinical guideline developers, research funders, journal editors, regulators and trial registries to act as advocates to ensure that their use is encouraged and supported.

### Study overview

The objective of the CONSENSUS (Squamous Cell CarcinOma of the OropharyNx: Late PhaSE CliNical TrialS; Core OUtcomeS) Study is to develop a core outcome set for OPSCC clinical trials. This protocol presents the methodology to be used.

The first stage of the study will establish the current standard of outcome reporting in clinical trials through a systematic review of OPSCC randomised controlled trials (RCTs). The second stage will involve qualitative interviews with OPSCC patients and carers to establish which outcomes are important to these key stakeholders. The third part of the study will employ an iterative consensus technique known as a Delphi study. We expect that different stakeholder opinions about which outcomes should be measured in clinical trials in OPSCC will converge to achieve consensus on the outcomes in the OPSCC core outcome set.

## Methods/Design

CONSENSUS is a mixed methods study. The Delphi questionnaire will be developed using a comprehensive list of outcomes identified from a systematic review, and outcomes identified from qualitative interviews with OPSCC patients and their carers.

Ethical approval for this study was granted in the UK by the Liverpool Central Research Ethics Committee (reference 12/NW/0708). Approval at the University of Texas MD Anderson Cancer Center (Houston, TX, USA) was provided by the Institutional Review Board (protocol number 2013-0285).

### Systematic review

A systematic review will identify which outcomes are reported in phase III RCTs of interventions for the treatment of OPSCC. This review will be limited to English language studies published in the last 10 years.

### Search strategy

A broad-based search strategy will be used to identify all published OPSCC and squamous cell carcinoma of the head and neck (SCCHN) RCTs. This will be applied to PubMed, Embase and the Cochrane Central Register of Controlled Trials (CENTRAL) from 1 January 2003 to 14 May 2013 (Appendix A).

### Types of studies and interventions

Any phase III RCT of a treatment for OPSCC with curative intent will be included. Only SCCHN trials that comprise patients with OPSCC will be included.

### Types of participants

Adults aged over 18 years with OPSCC.

### Exclusion criteria

Studies without OPSCC patients, studies involving patients with recurrent or metastatic disease, and studies of interventions for the treatment of the side-effects of treatment, such as xerostomia.

### Eligibility of studies

Two reviewers (AW and a second reviewer) will independently assess the identified records. A large number of records will be identified by our search strategy, therefore studies will be excluded in three phases. All identified study titles will be reviewed and ineligible studies excluded. Studies that are eligible from the title or for which there is uncertainty will have their abstracts reviewed in the second phase. Again, studies that are eligible or for which there is uncertainty will have their full papers reviewed in the third phase.

In order to ensure accuracy of exclusion, a proportion of all included/excluded titles, abstracts and full papers will be reviewed by two senior authors (TMJ and CTS).

A proportion of the studies excluded by title will have their abstracts reviewed. If more than 1% of these studies are found to be eligible then a further proportion of the same amount will be reviewed, until 1% or less of further studies in the re-review sample are eligible.

### Data extraction

Outcomes will be identified within the methods and results section of each paper, and for each outcome we will assess how it was defined and measured, the number of participants in which it was measured, whether it was measured using a validated tool, and whether it was stated in both the methods and results. The individual outcomes will be categorised under broader outcome domains.

### Data analysis

All eligible studies will be tabulated, and the identified outcomes presented along with their definitions and method of measurement. We also wish to identify any heterogeneity in the definitions of outcomes and the way in which they are measured. Scoping searches have suggested that outcomes with the same name are often defined or measured in different ways. We therefore wish to establish whether outcomes are defined and, if they are, whether there is heterogeneity in these definitions between studies which would make comparison of these outcomes between trials more difficult, if not impossible.

The identified outcomes will be categorised under broader outcome domains which will be decided upon by the study team. For functional outcomes, this process will be guided by the International Classification of Functioning, Disability and Health (ICF) categories for head and neck cancer [15].

### Qualitative interviews

The objectives of the qualitative part of the study are to establish which outcomes are valued by patients and carers, and which outcomes they feel should be included in a core outcome set. The journey from diagnosis through treatment is often a turbulent one for patients with OPSCC and qualitative methods will be particularly useful in helping us to understand their experiences of treatment. Spouses will also be interviewed as they almost universally become carers to their partners during this time and can provide an expert witness account of events whilst helping to contextualise the importance of different outcomes.

### Participants

Patients with OPSCC and their carers will be recruited to participate in qualitative interviews, from two centres in the UK and one in the USA. A carer is defined as a family member or spouse who provides informal care to the patient during their treatment and recovery. Patient and carer participants will be recruited from SCCHN survivorship clinics. Patient participants will be adults, aged 18 to 100 years, who are English speaking and up to 5 years post-treatment for OPSCC. Carers of eligible patient participants will also be invited to interview.

Eligible patients will be recruited from the outpatient clinic in a chronological order to avoid the risk that clinic staff will ‘cherry-pick’ patients who they anticipate will present a favourable account of their experiences. We will monitor the clinical and socio-demographic characteristics of our patient sample, and ensure maximum diversity in terms of the following characteristics: age, gender, sub-site, stage, HPV status, treatment modality and length of time since treatment. Maximum diversity sampling, a type of purposive sampling, is widely used in qualitative research with the aim of accessing a range of perspectives on the topic under investigation [16]. We will interview NHS patients in the UK, for whom care is provided free at the point of delivery and patients attending the University of Texas MD Anderson Cancer Center, which is a fee-paying institution. The UK and US groups will differ in their socio-economic circumstances, thus the sample encompasses diversity on this characteristic, which is well-recognised as having a significant impact on outcomes in SCCHN patients [17].

HPV status will not be available for patients whose diagnosis and treatment preceded testing for HPV. However, we will endeavour to recruit a mix of HPV-positive and HPV-negative patients.

We expect to recruit around 30 patients and carers in total, although this may change depending on early analyses.

### Interview format

A semi-structured interview format will be used. Participants will be required to provide informed consent prior to interview. Conversation will be guided by a prompt guide of open-ended questions on topics such as diagnosis and treatment, and the effect the cancer and treatment had on participants’ lives, both at the time of treatment and at the time of the interviews. Therefore, whilst the interview will be conversational in nature, discussion will be directed towards identifying outcomes of importance to patients and carers. Towards the end of the interviews we will directly ask participants which outcomes they think are most important and should be measured in clinical trials. Key to this interview technique is that it allows participants to explain their experience of the illness and treatment in their own terms, and to raise and focus on the aspects of their journey that are most important to them.

### Interview analysis

In line with the principles of qualitative research, the analytical process will begin during data collection, as the data already gathered are analysed to inform the ongoing data collection. This will allow the study team to go back and refine questions, develop hypotheses, and pursue emerging avenues of inquiry in further depth in subsequent interviews [18].

We will take an interpretive approach, informed by the principles of the constant comparative method and by several procedural steps to ensure the quality of the analysis [19]. This involves an inductive process of identifying analytical categories as they emerge from the data (developing hypotheses from the ground or research field upwards rather defining them *a priori*). The analysis of patients’ and carers’ accounts will initially proceed in parallel, but related, courses. We shall analyse within each group for common themes, such as what is important to patients/carers, and how their lives have been affected by the cancer and treatment [20]. As the analysis develops we will compare across the different groups to identify convergences and divergences. We will examine how participants present their accounts as well as the content of the interviews. That is, we will not simply take participants’ accounts at face value.

We will compare data across the different groups (age, gender, sub-site, stage, HPV status, treatment modality, socio-economic status and length of time since treatment) and analyse for patterns in how these characteristics shape participants’ priorities following cancer treatment. As the principal investigator (PI), AW will lead a process of ‘cycling’ between the developing analysis and new data, and the complete team will develop and ‘test’ the analysis by periodic discussion of transcripts and reports on the developing analysis. Initially, each transcript will be read several times by AW before developing open codes to describe each relevant unit of meaning. Initial open coding will occur at multiple levels, from detailed descriptions of experiences line by line, to the general stance participants take towards different aspects of their lives. Through comparison within and across the transcripts the open codes will gradually be developed into theoretical categories and subcategories, to reflect and test the developing analysis.

The categories will be organised into a framework to code and index the transcripts using NVivo qualitative data analysis software (version 10, 2012; QSR International Pty Ltd, Victoria, Australia). The framework categories will be continually checked and modified to ensure an adequate ‘fit’ with the data, whilst also accounting for variant or exceptional cases. The categories and the assignment of data to them will be reviewed by a second member of the project team.

### The Delphi method

In order to gather opinion and to reduce the number of outcomes to a priority list for consideration in future OPSCC clinical trials we will use the Delphi method. This was originally developed by the RAND Corporation in the 1950s in order to forecast the influence of technology on warfare [21]. It is an iterative consensus technique which comprises sequential questionnaires answered anonymously by a panel of participants with relevant expertise [6].

Questioning takes place in rounds, and after each round of questions, an anonymous summary of the responses is fed back to the group. Individual participants may then decide to keep their original answers or to change their opinion in the subsequent round of voting. The advantage of this approach is that it is not face-to-face, and therefore avoids the problem of more vocal or apparently senior participants dominating the group discussion and therefore influencing others’ voting.

In general, the range of answers decreases and the group converges towards a consensus opinion over the course of several rounds. The process terminates after a pre-defined stop criterion, which for this study will be whichever occurs first: reduction of the long list of outcomes to ten or less; or on completion of the second round of voting.

## Synthesis of outcomes into Delphi questionnaire

### Outcomes from systematic review

The outcomes highlighted in the systematic review will be categorised under broader outcome domains, and presented in the questionnaire as such.

### Outcomes from interviews

Identifying measureable outcomes from the interview transcripts will be a more complex process. While some outcomes are likely to be obvious, the context must always be borne in mind, and it is important that our interpretation of the interview transcripts avoids either overlooking outcomes of importance to participants or misrepresenting them. For example, how extensively a participant talks about a particular aspect of their life is not necessarily indicative of its importance to them, since patients may talk about highly significant issues in a seemingly brief and casual manner as a way of managing their emotions. We will begin by analysing transcripts to identify experiences and priorities that map straightforwardly to obviously measurable outcomes. Examples include mouth dryness, difficulty swallowing and fatigue. Secondly, where a participant has described an experience or issue that was significant for them, the study team will attempt to map this to a measurable outcome. An example of this could be anxiety about survivorship clinic appointments, which is likely to be an expression of a patient’s anxieties about recurrence and survival. One researcher (AW) will be responsible for making these interpretations, and two researchers (AW and BY) will review a proportion of the outcomes and the supporting evidence from the interview transcript to scrutinise and ‘test’ the plausibility of the interpretations involved in translating participants’ accounts of their experiences and priorities into outcomes. Any uncertainties and a proportion of all outcomes derived through this process will be discussed with the whole study team (AW, CTS, BY, TMJ).

Patient interviews will be given primacy over the carer interviews in the analysis and derivation of outcomes. The carer acts as a witness to the patient’s experience and therefore their accounts will be used largely to illuminate our analysis of the patient accounts. For example, we will identify how far carers confirm or refute patient accounts; where divergences arise we will return to the patient accounts to identify the reasons for these or reconsider our interpretations as appropriate. The interview data will also be used to inform the presentation and labelling of outcomes, particularly for patients, in the Delphi study.

### Participants

The Delphi study will survey individuals with a stake in clinical trials for OPSCC. This includes OPSCC patients and their carers, and healthcare professionals involved in the management of patients with OPSCC, namely, medical and surgical oncologists, speech and language therapists (SLTs), and head and neck clinical nurse specialists (CNSs). It is important that a proportion of our clinician participants are also involved in research or clinical trials; therefore, we will recruit medical and surgical oncologists and SLTs from a list of those involved in a multicentre SCCHN trial that is ongoing and coordinated by our unit, and CNSs through personal contacts and professional bodies.

In the UK, patient and carer interviewees will be approached about participation in the Delphi study at the time of the interview. Additional patient and carer participants will be identified within survivorship clinics. The Delphi questionnaire will be included with a postal invitation, and a follow-up telephone call will be made 7 days following postage to confirm that the questionnaire was received. Clinician participants will be invited by email to participate in an online Delphi questionnaire and a reminder email will be sent after 7 days. The invitation will clearly state the importance of completing all rounds of the Delphi study. Attrition is more likely in participants with minority opinions and this can lead to an overestimation of the degree of consensus in the final results [6]. It will be clearly stated that outcomes important to that individual may not be included in the final core outcome set.

### Definition of consensus

This must be defined upfront to avoid consensus being defined in a way that could bias the results towards the beliefs of the research team. For an outcome to be included in the core outcome set there must be majority agreement of the critical importance of an outcome, and minority agreement that the outcome is not important. Conversely, for an outcome to be excluded there must be majority agreement that the outcome is not important, and only minority agreement that it is critically important. These judgements are based on GRADE Working Group recommendations and are discussed in further detail below [22,23].

### Statistical considerations

There are no recommendations for the number of participants to include in a Delphi study. In a systematic review by Sinha *et al*. of studies using the Delphi method in core outcome set development, the number of participants ranged from 13 to 222 [6]. We have a heterogeneous group of participants and therefore our sample size will need to be slightly larger to account for the likely diversity of opinions [24]. We aim to recruit around 30 patients and carers, and 30 clinicians, who will be medical and surgical oncologists, SLTs, and head and neck CNSs.

#### ***Voting***

In other Delphi studies a ‘blank sheet’ round has been used to canvas participant opinion and generate a comprehensive and inclusive list of candidate outcomes. However, in conducting the systematic review and interviews we have effectively completed this process.

### Round 1

In round 1 of this Delphi study we will ask participants to rate each of the outcomes using the GRADE process, which suggests a 9-point scale (1 to 9) to rank their importance [23]. There will also be room for participants to add additional outcomes and to comment on why they have ranked outcomes as they have. Rankings of 7 to 9 indicate outcomes of critical importance, ratings of 4 to 6 represent outcomes that are important but not critical, while ratings of 1 to 3 are items that are deemed to be of limited importance. All outcomes will be carried through to the second round with first round scores displayed for each outcome. Consensus to carry an outcome through to the core outcome set will be defined as more than 70% of participants scoring its importance as 7 to 9 and less than 15% scoring it as 1 to 3. Additional outcomes will be coded by the study team using the same methods as for the interview and systematic review. Feedback will be analysed using software adapted from another Delphi study.

### Round 2

The number and percentage of participants that allocated each score will be presented to participants. However, feedback to each group will be randomised such that some participants receive only their group’s voting and others receive both groups’ voting. Participants will then be asked to rate the outcomes again, using the 9-point scale.

### Analysis of voting

The opinions of different groups can be analysed either together or separately. Differences in opinion can be accounted for by having separate panels for different stakeholder groups [25]. It is not clear at this stage whether patients and carers will have different opinions to healthcare professionals. Macefield *et al*., in using a Delphi study to develop a core outcome set, demonstrated that the way in which feedback is delivered to participants affects the voting in subsequent rounds [26]. Feedback was randomised such that a proportion of both stakeholder groups received feedback from both stakeholder groups, and not just their own group. This altered voting, especially by the clinician group. We will randomise feedback such that half of the patient/carer group will receive results from the patients’/carers’ first round of voting, and half will receive results from patients/carers and clinicians. Similarly, half of the clinicians will receive results of the clinicians’ voting in the first round, and half will receive feedback from both patients/carers and clinicians. It is important to ensure that we have similar numbers between the panels for every round so that the final consensus is not numerically dominated by one group’s responses. As recommended by Sinha *et al*., we will report a measure of the distribution of scores for each outcome considered in the final round [6]. This is because cut-off scores, used in most studies, do not describe how strongly the minority feel, and so an apparent consensus could actually be masking major disagreement within the group [27].

### Consensus meeting

After round 2, the remaining outcomes will be reviewed. If consensus has not been reached or there is significant disagreement between the groups, we will conduct a face-to-face meeting of 15 Delphi participants who are key stakeholders (patients, carers, medical and surgical oncologists, SLTs, and CNSs) to resolve any disagreement, and discuss the remaining outcomes and their application within clinical trials. By the end of this process we should have identified ‘what’ outcomes to measure, although we may well not be clear on the best way of measuring these.

## Discussion

A core outcome set for OPSCC will improve the conduct, reporting and contribution of clinical trials to the existing body of evidence for OPSCC in the published literature. We believe that establishing which outcomes to measure takes priority over establishing how to measure these outcomes as any recommendation regarding a specific instrument may well become outdated.

As stated previously, a core outcome set will only have impact if it is consistently implemented in trials. We must actively engage with trialists, regulators, and those that fund and publish trials to ensure that our core outcome set is used, and that there are incentives to use it, not just in the UK but in the rest of the academic world.

## Trial status

We are preparing to recruit participants to the Delphi study.

## Appendix A

### Search strategies for systematic review

PubMed search strategy: 1 January 2003 to 14 May 2013

1. “Oropharyngeal Neoplasms”[Mesh]

2. (“Head and Neck Neoplasms”[Mesh:NoExp])

3. “Otorhinolaryngologic Neoplasms”[Mesh:NoExp]

4. “Pharyngeal Neoplasms”[Mesh:NoExp]

5. “Neoplasms”[Mesh]

6. (cancer* OR carcinoma* OR neoplas* OR tumor* OR tumour* OR malignan* OR SCC)

9. (#5 OR #6)

10. “Oropharynx”[Mesh]

11. (oropharyn* OR mesopharyn* OR tonsil* OR “head and neck” OR “head neck” OR “head-neck” OR “head-and-neck” OR “tongue base” OR “soft palate”)

12. (#10 OR #11)

13. (#9 AND #12)

14. (HNSCC ORSCCHN OR OP-SCC OR OPSCC)

15. (#1 OR #2 OR #3 OR #4 OR #13 OR #14)

16. ((((Randomized Controlled Trial[ptyp])) OR ((Controlled Clinical Trial[ptyp])) OR ((Clinical Trial[ptyp])) OR (“Clinical Trials as Topic”[Mesh]) OR (“Clinical Trials, Phase III as Topic”[Mesh]) OR (“Clinical Trials, Phase IV as Topic”[Mesh]) OR (“Controlled Clinical Trials as Topic”[Mesh]) OR (“Clinical Trial”[Publication Type]) OR (“Controlled Clinical Trial”[Publication Type]) OR (“Clinical Trial, Phase III”[Publication Type]) OR (“Clinical Trial, Phase IV”[Publication Type]) OR (“Multicenter Study”[Publication Type]) OR (“Multicenter Studies as Topic”[Mesh]) OR (“Random Allocation”[Mesh]) OR (“Double-Blind Method”[Mesh]) OR (“Single-Blind Method”[Mesh]) OR (“Cross-Over Studies”[Mesh]) OR (“Placebos”[Mesh]) OR (controlled[tiab] AND (trial[tiab] OR trials[tiab] OR study[tiab] OR studies[tiab])) OR (blind[tiab] OR blinding[tiab] OR blinded[tiab] OR mask[tiab] OR masking[tiab] OR masked[tiab] OR placebo[tiab] OR placebos[tiab] OR rct[tiab] OR random[tiab] OR randomised[tiab] OR randomized[tiab] OR randomly[tiab] OR randomisation[tiab] OR randomization[tiab]) OR (factorial[tiab]) OR (divided[tiab] AND (group[tiab] OR groups[tiab])) OR (crossover[tiab]) OR (“cross over”[tiab]) OR (multicentre[tiab] OR multicentred[tiab] OR multicentric[tiab]) OR (versus[ti] OR vs[ti]) OR (“treatment arm”[tiab]) OR (“phase III”[tiab] OR “phase three”[tiab] OR “phase 3”[tiab]) OR (“latin square”[tiab]) NOT ((“Animals”[Mesh] OR mouse[ti] OR mice[ti] OR pig[ti] OR pigs[ti] OR rat[ti] OR rats[ti] OR rabbit*[ti]) NOT ((“Animals”[Mesh] OR mouse[ti] OR mice[ti] OR pig[ti] OR pigs[ti] OR rat[ti] OR rats[ti] OR rabbit*[ti] OR cadaver[ti] OR cadavers[ti]) AND (“Humans”[Mesh])))))

17. (#15 AND #16)

18. ““Cochrane Database Syst Rev”“[Journal]

19. (“systematic review” OR “meta analysis”)

20. (#19 OR #18)

21. (#17 NOT #20)

Cochrane Central Register of Controlled Trials (CENTRAL): 1 January 2003 to 14 May 2013

1. MeSH descriptor: [Oropharyngeal Neoplasms] explode all trees

2. MeSH descriptor: [Head and Neck Neoplasms] this term only

3. MeSH descriptor: [Pharyngeal Neoplasms] this term only

4. MeSH descriptor: [Otorhinolaryngologic Neoplasms] this term only

5. MeSH descriptor: [Neoplasms] explode all trees

6. cancer* OR carcinoma* OR neoplas* OR tumor* OR tumour* OR malignan* OR SCC

7. #5 OR #6

8. MeSH descriptor: [Oropharynx] explode all trees

9. oropharyn* OR mesopharyn* OR tonsil* OR “head and neck” OR “head neck” OR “head-neck” OR “head-and-neck” OR pharyn* OR “tongue base” OR “soft palate”

10. #8 OR #9

11. #7 AND #10

12. HNSCC OR SCCHN OR OP-SCC OR OPSCC

13. #1 OR #2 OR #3 OR #4 OR #11 OR #12

Embase: 1 January 2003 to 14 May 2013

1. Exp Oropharynx tumor/

2. Pharynx cancer/

3. Neoplasm/

4. (cancer* OR carcinoma* OR neoplas* OR tumor* OR tumour* OR malignan* OR SCC).tw.

5. #3 OR #4

6. exp OROPHARYNX/OR exp OROPHARYNX CANCER/OR exp OROPHARYNX CARCINOMA/

7. (oropharyn* OR mesopharyn* OR tonsil* OR “head and neck” OR “head neck” OR “head-neck” OR“head-and-neck” OR pharyn* OR “tongue base” OR “soft palate”).tw.

8. #6 OR #7

9. #5 AND #8

10. (HNSCC OR SCCHN OR OP-SCC OR OPSCC).tw.

11. #1 OR #2 OR #9 OR #10

12. (random* OR factorial* OR placebo* OR assign* OR allocat*).mp. OR crossover*.tw. [mp = title, abstract, subject headings, heading word, drug trade name, original title, device manufacturer, drug manufacturer, device trade name, keyword]

13. (cross adj over*).tw.

14. (cross adj over*).tw.

15. ((blind* OR mask*) and (single OR double OR triple OR treble)).tw.

16. (treatment adj arm*).tw.

17. (control* adj group*).tw.

18. (phase adj III).mp. OR three.tw. [mp = title, abstract, subject headings, heading word, drug trade name, original title, device manufacturer, drug manufacturer, device trade name, keyword]

19. (versus OR vs).tw.

20. rct.tw.

21. CROSSOVER PROCEDURE/

22. DOUBLE BLIND PROCEDURE/

23. SINGLE BLIND PROCEDURE/

24. RANDOMIZATION/

25. PLACEBO/

26. exp CLINICAL TRIAL/

27. PARALLEL DESIGN/

28. LATIN SQUARE DESIGN/

29. #12 OR #13 OR #14 OR #15 OR #16 OR #17 OR #18 OR #19 OR #20 OR #21 OR #22 OR #23 OR #24 OR #25 OR #26 OR #27 OR #28

30. #11 AND #29

31. limit #30 to human

32. “systematic review”.tw.

33. #31 NOT #32

## Abbreviations

CENTRAL: Cochrane Central Register of Controlled Trials; CNS: Clinical nurse specialist; COMET: Core Outcome Measures in Effectiveness Trials; CRT: Chemoradiotherapy; HPV: Human papillomavirus; HPV-16: Human papillomavirus type 16; HTA: Health Technology Assessment; ICF: International Classification of Functioning, Disability and Health; NHS: National Health Service; NIHR: National Institute for Health Research; OMERACT: Outcome Measures in Rheumatology; OPSCC: Oropharyngeal squamous cell carcinoma; PI: Principal investigator; PRO: Patient-reported outcome; RA: Rheumatoid arthritis; RCT: Randomised controlled trial; RT: Radiotherapy; SCCHN: Squamous cell carcinoma of the head and neck; SLT: Speech and language therapist.

## Competing interests

The authors declare that they have no competing interests.

## Authors’ contributions

AW conceived and designed the study, and wrote the manuscript. CTS conceived and designed the study, and provided critical revision of the manuscript. BY conceived and designed the study, and provided critical revision of the manuscript. TMJ conceived and designed the study, and provided critical revision of the manuscript. All authors read and approved the final manuscript.
